# How Digital Food Affects Our Analog Lives: The Impact of Food Photography on Healthy Eating Behavior

**DOI:** 10.3389/fpsyg.2021.634261

**Published:** 2021-04-06

**Authors:** Tjark Andersen, Derek Victor Byrne, Qian Janice Wang

**Affiliations:** ^1^Food Quality Perception and Society, Department of Food Science, Faculty of Technical Sciences, Aarhus University, Aarhus, Denmark; ^2^Sino-Danish College (SDC), University of Chinese Academy of Sciences, Beijing, China

**Keywords:** grounded cognition, food photography, social media, commensality, food intake, food porn

## Abstract

Obesity continues to be a global issue. In recent years, researchers have started to question the role of our novel yet ubiquitous use of digital media in the development of obesity. With the recent COVID-19 outbreak affecting almost all aspects of society, many people have moved their social eating activities into the digital space, making the question as relevant as ever. The bombardment of appetizing food images and photography – colloquially referred to as “food porn” – has become a significant aspect of the digital food experience. This review presents an overview of whether and how the (1) viewing, (2) creating, and (3) online sharing of digital food photography can influence consumer eating behavior. Moreover, this review provides an outlook of future research opportunities, both to close the gaps in our scientific understanding of the physiological and psychological interaction between digital food photography and actual eating behavior, and, from a practical viewpoint, to optimize our digital food media habits to support an obesity-preventive lifestyle. We do not want to rest on the idea that food imagery’s current prevalence is a core negative influence *per se*. Instead, we offer the view that active participation in food photography, in conjunction with a selective use of food-related digital media, might contribute to healthy body weight management and enhanced meal pleasure.

## Introduction

Obesity continues to be a major global problem for individual welfare (see Reilly, 2003) and a burden on national health care systems (see Wang et al., 2011). By 2030, half of the United States population will be obese (The Lancet Diabetes Endocrinology, 2020). In Europe, the obesity rate has been linearly increasing ever since the 1970s, without any indication of slowing down (WHO, 2016). Observational studies have shown that watching TV is associated with being overweight (e.g., Gore et al., 2003; Halford et al., 2004; for a review, see Boulos et al., 2012). Simultaneously, food is becoming an increasingly popular topic both in classical forms of media, such as cooking books and TV, as well as in new digital media formats on the internet, such as YouTube, Instagram, and other social media platforms (Petit et al., 2016; Spence et al., 2016). In 2014, Google reported food and cooking to be the fastest growing topic on YouTube (Kantchev, 2014). In a recent survey of United States adults, 88% reported to eat while looking at a screen, and on average respondents reported having only five screen-free meals a week (Anderer, 2019). In general, the rise in food media’s popularity may be a consequence of the decline of time dedicated to food preparation (also Prince, 2014; Spence et al., 2016), potentially leaving people psychologically unfulfilled and hungry (cf. also Murray and Vickers, 2009; cf. Dohle et al., 2014).

Mukbang, originating in South Korea, is a modern participatory digital food trend in which a host broadcasts eating large quantities of food while interacting with the audience (Pereira et al., 2019; Anjani et al., 2020). On Instagram and direct digital communication, it is also increasingly popular to participate actively in food content creation instead of mere passive consumption by taking photos of one’s meals and sharing it with followers, friends, and family. To illustrate, the number of photos uploaded to Instagram every minute has increased by 17% from 2017 to 2019 (DOMO, 2017, 2019). Such active participation is also known as “user-generated content” (UGC).

Especially among adolescents, digital media (and social media specifically) have all but overtaken the traditional media of print and TV (Twenge et al., 2019). United States 12th graders spent approximately twice as much time online in 2016 than they did in 2006. Conversely, the share of 10th graders who read print every day has declined from 60% in the 1970s to 16% in 2016. Similarly, daily TV consumption has declined by an hour from the early 1990s to 2016. Research has yet to completely map out the implications of the ascend of these new and participatory digital media forms, including if and how they might affect the continued rise of obesity. Naturally, finding ways to utilize these media forms to facilitate, rather than hinder, healthy eating is of interest.

The recent outbreak of COVID-19 has increased this trend toward digitalization further. Among the chief restrictions caused by the pandemic have been limitations on people’s social lives. A recent survey during the first wave of the pandemic around April 2020 found that social activities had decreased between 46.7 and 58%, and overall life satisfaction had decreased by 30.5% (Ammar et al., 2020b). Eating behavior and physical activity levels were also adversely affected, with a reported decrease in total weekly activity minutes by 33%, higher reported meal frequency, snacking, “out-of-control” eating, and more unhealthy food choices (Ammar et al., 2020a). Somewhat expected, the number of individuals who used digital technology for social purposes had increased by 24.8% (Ammar et al., 2020b). Eating is, in large part, a social affair (cf. Herman, 2017), therefore, it seems natural to assume that a non-trivial share of newly-digitized social interaction involved food. Indeed, a recent survey of people’s motivations and experiences of lockdown-related online dining found that people gathered mainly for social reasons (Ceccaldi et al., 2020). The experience itself was reported as rather insufficient. We have possibly witnessed the largest experiment in digital commensality to date (cf. Spence et al., 2019). Hence, the question of how digital media affect eating seems more relevant than ever.

### Food Photography

The term “food porn” was coined in the late 1970s to describe mouth-watering images of food that are “sensationally out of bounds of what food should be” (McBride, 2010). In other words, food images become pornographic when they showcase a visual decadence that is *entirely* removed from food’s primary function – nourishment. The term has caught on and is nowadays used more generally for online viewing and sharing of appetizing food images (see Petit et al., 2016). However, not all scholars agree on this definition or even the notion that food photography could be pornographic in principle. Tooming (2021) conceptualizes pornography as a means to obtain sexual release. According to this definition, then, food images cannot be pornographic because they cannot afford the release, i.e., satiation.^1^

On the question of motivation, Tooming questions the precise source of pleasure when viewing food photography. The author, at least in part, dismisses anticipation as the source of pleasure in viewing food photography, as desiring the literally depicted food is both irrational (it is neither accessible and probably does not even exist anymore) and the activity does not seem to cause people to replicate the food, either (Prince, 2014). Neither is the pleasure purely visual. In his estimation, the pleasure in viewing food images is best described as *“reality-independent [gustatory] imaginings.”* These “imaginings” are independent from reality insofar as they are not entirely bound to the actually depicted food (which might be a stylized mixture of inedible substances, see Chapin, 2016). We will return to these notions when discussing relevant experimental evidence.

While also defending the notion that food photography *could* have artistic merit, Tooming admits that most food photography on social media – maybe especially the popular ones – would probably not be considered art, or at least not good art, for it mostly appeals to universal, instead of learned or acquired, sensitivities (cf. Matthen, 2015). Regarding social media more generally, it is important to note that food companies invest heavily into the platforms and content to either influence consumers directly or gain detailed behavioral insight (Lewis, 2018). Here also, automated content contributions from bots play a role (cf. generally Daniel et al., 2019). While certainly important, we will not pursue these aspects of online food content quality in the review at hand.

The number of digital photos taken has increased over the past decades. On the year 2000, a reported 80 billion photos were taken, and by 2015, this number had grown to a trillion (Heyman, 2015). The increase is reportedly due to the spread of camera-equipped smartphones, with 75% of 2015’s photos taken with such devices. According to a poll, 81% of the United States population owned a smartphone in 2019 (Pew Research Center, 2019). We assume that the figures in other developed countries are similarly high.

The explosion of content on food-related peer-review websites such as TripAdvisor and Yelp (Melumad et al., 2019) would suggest that food photography – sometimes also referred to as “foodtography” (Coary and Poor, 2016) – is a widely prevalent activity. However, it can be controversial. Multiple restaurants, including well-known fine dining establishments, restrict food photography (Ensor, 2013; Stapinski, 2013; Willis, 2017). The restrictions vary and can include anything from a ban on flash-usage to disallowing any food or restaurant interior photography. There seem to be multiple issues. First, food photography causes an interruption in meal procedure and leaves the food to cool down, diminishing the experience of the diner herself. Second, photographing with a flash disrupts other’s meal experience. Third, photos provide only a partial representation of the food – *“a picture on a phone cannot possibly capture the flavors”* (Willis, 2017) – resulting in inferior marketing for the restaurant. High-end chefs’ critical stance toward food photography has not changed in recent years (Rawlinson, 2020).

A recent scientific publication by Yong et al. (2020) suggests a more mild prevalence of food photography. Investigating a group of healthy students (18–30 years old) at the University of Singapore under free-living conditions, the researchers report an average meal-time photography rate of about 5%, measured across a total of about 7,000 recorded meals. Meanwhile, only 23% of all study participants partook in *any* meal-time photography, who, in turn, photographed 16% of their meals. These findings seem to suggest that a minority of power-users capture most of the photographic content, at a rate of about one out of six meals. The employed Experience Sampling methodology reached a response rate of 76%, lending credibility to the results. Unfortunately, the study did not report the context of meal photography. Thus, it is impossible to draw any conclusions on whether the widespread reports of food photography’s ubiquity are an overestimation or merely a matter of context.

One aspect that food photography is used for is cross-cultural communication. Food is an obvious human necessity. This fact makes food universally relatable and, consequently, a good vehicle for cross-cultural empathy (cf. Woolley and Fishbach, 2017). For example, people living away from their childhood region or country may feel nostalgic in relationship to specific food posted online (Connolly, 2015). While people from other cultures may not have any particular memories tied to the same food, they nevertheless can recognize the sentiment and empathize with the person posting about the nostalgic food experience. More generally, food is associated with and implied in all aspects of the human experience (Ibrahim, 2015). By publicly sharing everyday food images, people allow for a deep and intimate look into their private lives, juxtaposing the private and public spaces. In this private and public hybrid space, cultural exchange is enhanced (Ibrahim, 2015). It seems not surprising, then, that these authors emphasize that “food is more than just fuel for bodies” (see also Connolly, 2015; Ibrahim, 2015).

### Aim

This review aims to provide an overview of the research related to the interaction between everyday food photography habits and healthy eating behavior, suggest potential opportunities for future research as well as practical application. In this review, we will look at the following three food photography habits:

1. *Viewing* digital food-content, e.g., viewing others’ food photography on social media;
2. *Creating* content, i.e., users engaging themselves in food photography; and
3. *Sharing* such content with other people.

We operationally define eating behavior as food choice and intake (Eertmans et al., 2001). With healthy eating, we refer to choosing nutrient-dense food in portion sizes supportive of healthy body function and level of body fat.

**Figure 1:**
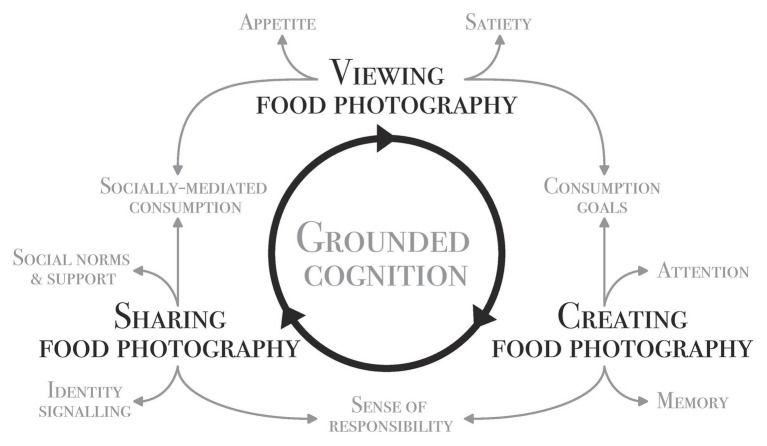
Schematic representation of the digital food photography habits cycle and related influences.

Under these definitions, we are neither exhausting all possible aspects of digital interaction with food in general, nor food photography in particular. For example, we will not cover in detail the pleasure of viewing food photography for its own sake, i.e., independently of an eating context. We will also not cover aspects of the social media landscape *per se*, e.g., market dynamics or incentives. Interested readers are referred elsewhere (cf. Labrecque et al., 2013; e.g., Lewis, 2018). Finally, we will not cover digital technology generally as a source of distraction (Oldham-Cooper et al., 2011; see, e.g., Teo et al., 2018). Instead, our focus will be on the direct interaction between viewing, creating, and sharing digital food photography and actual eating behavior at the physiological and psychological levels. Figure 1 provides a schematic overview of the three activities and their relationship with each other and the research themes covered in this work.

The review is structured into two main sections. In the first, “State of the Art,” we review and discuss direct research relating *viewing*, *creating*, and *sharing* food photography and eating behavior. In the second, “Future Perspectives,” we will connect analogous and related research and hypothesize about other interactions between food photography and eating behavior, yet to be studied directly. The section will also include practical take-away points to guide the use of food photography in our analog lives.

### Theoretical Underpinning: Grounded Cognition and Mental Simulation

Before discussing how each aspect of food photography might influence us, it is worth pointing out the cognitive mechanisms underlying behavior. Grounded cognition theories assume that cognition is fundamentally grounded in situated action, bodily states, and (mental) simulation (Barsalou, 2008). Action is inherently goal-directed and situated in the environment, which we perceive primarily in terms of affordances and constraints in relation to the goal (Gibson, 1979). Cognitive processes are affected by internal and external bodily states, e.g., feelings of anger or a cold environment. Mental simulations are re-enactments of perceptions in their respective brain areas, e.g., imagery in the visual cortex. The three pillars of grounded cognition are tightly connected and interdependent.

The concept of *modal* representations of knowledge is central in cognition. This means that the *“brain areas representing knowledge for a particular category are those typically used to process its physical instances”* (Simmons et al., 2005). Especially in this point grounded theories stand in opposition to classical theories of cognition, which claim that cognition works mainly on *amodal*, abstract symbols, with embodied and cross-modal effects only being peripheral or epiphenomenal (Barsalou, 2008). There appears to be no central “simulation area” in the brain; instead, multiple areas jointly produce these mental images. The activation of these brain areas can lead to subsequent physiological responses downstream, including desire to consume and satiation (for a review, see, e.g., Krishna and Schwarz, 2014; more recently, Papies et al., 2020). To quote Christian et al. (2016, p. 85): *“The pathway from simulation to consumption rests on the fact that mental imagery is facilitated by reactivation of the sensorimotor systems that support perception and action.”* It might be worth pointing out that, while very much related, mental simulation and imagery are not the same. The former is an automatic and implicit process, while the latter is deliberate and conscious (Simmons et al., 2005). Either way, we see mental simulation and imagery as essential cognitive mechanisms in understanding the effect of viewing, creating, and sharing food photography.

Sociality is fundamental for humans, and we use a whole range of cognitive processes to successfully navigate the social fabric (see Barsalou, 2008). For example, based on others’ visible cues and mental simulation, we infer their goals and affective states. The visual cues themselves activate so-called *mirror neurons*, which are an essential component in the inference process. Besides their role in empathy, mirror neurons are also implied in imitation-based activities, such as learning from others and social coordination. The fact that we have evolved dedicated brain structures to optimally pick-up on social cues illustrates their importance for regulating behavior.

## State of the Art

### Viewing Food Photography

#### The Biological Link Between Viewing Food Photography and Behavior

As alluded to in the previous section on grounded cognition theories, the modal representation of knowledge implies that knowledge of categories is represented in the same brain areas as those activated when engaging with the physical instances. This is also the case for food. Thinking about and seeing food depictions activates the same gustatory system as seeing real food (Simmons et al., 2005). The brain essentially infers taste as well as taste and consumption reward – including energy content (Toepel et al., 2009; van der Laan et al., 2011) – based on the visual food cues. Accordingly, hunger is modulated on at the neural level (van der Laan et al., 2011).

Evolution may have evolved the human brain to be highly responsive to visual food cues, with obvious survival benefits for pre-historic man. In fact, a dedicated neural network for eating has been identified (Chen et al., 2016) and recent evidence suggests that attention is biased toward food compared to non-food cues (see Spetter et al., 2020). This bias has been demonstrated both behaviorally (Higgs et al., 2012, 2015; Rutters et al., 2015; Kumar et al., 2016; Kaisari et al., 2019) and at the neurological level (Spetter et al., 2020). The cognitive basis for the effect is 2-fold. Firstly, while thinking of anything – i.e., holding representations in working memory – guides perceptual awareness toward similar environmental stimuli, this effect is more pronounced for food stimuli, especially when high in energy content (Toepel et al., 2009), due to their higher affective value (cf. generally Zeelenberg et al., 2006). Secondly, food representations are held more efficiently in working memory than those of non-food items. Therefore, more working memory will be available to process new environmental stimuli. In combination, these findings indicate that thinking about food further primes attention for food cues.

One general moderator of the visual attentional bias toward food is hunger. When hungry, individuals exert a higher bias toward food cues in general, irrespective of reported liking for the depicted food (Piech et al., 2010; Davidson et al., 2018). While satiation leads to some general attenuation of the bias, it is also highly sensory-specific, and correlates with post-consumption changes in individual food’s pleasantness (cf. Rolls et al., 1981b; di Pellegrino et al., 2011). In fact, the satiation is sufficiently specific so that, for example, *ad-libitum* consumption of one type of sandwich only moderately decreases attentional bias for photographs of other types of sandwiches, and barely at all for photos of desserts (Davidson et al., 2018).

Individuals also differ in their proclivity to manifest biased food attention. For example, individuals scoring high in the trait restraint and low the trait disinhibition have a less of an attentional bias and also overall pay less attention to food cues (Higgs et al., 2015). A recent study showed that overweight and obese individuals engage in greater top-down attention modulation compared to normal weight individuals, which, in turn, was predictive of weight gain 1 year later (cf. Castellanos et al., 2009; Kaisari et al., 2019). A previous systematic review had similarly concluded that overweight and obese individuals show enhanced neural responses to food cues compared to lean individuals, particularly for energy-dense food (Pursey et al., 2014; cf. also Brunstrom et al., 2018).

The hormone ghrelin is a major mechanism linking visual food stimuli and eating behavior. In a classic paper, Wren et al. (2001) demonstrated in humans that exogenous ghrelin administration causes increases in both hunger and food intake. In the experiment, the blood-infused ghrelin led to a 28% increased energy intake at a subsequent buffet meal, compared with the saline control solution. In absolute terms, this increase amounted to an additional 300 kcal. Thus, ghrelin has become known as the “hunger hormone” (Hsu et al., 2016).

However, it might be more correct to refer to ghrelin as the “food anticipation hormone” (see Frecka and Mattes, 2008). For example, Frecka and Mattes (2008) showed that ghrelin blood concentrations are entrained to the habitual meal schedule and do not necessarily correlate with reported hunger levels. Researchers have implied the hormone in priming the gastro-intestinal system for food (Drazen et al., 2006). The connection between circulating ghrelin levels and food intake may, at least in part, be a learned response linking interoception and behavior (Hsu et al., 2016). More generally, ghrelin plays an important role in the regulation of food reward in the brain, *via* interaction with dopaminergic neurons (for a review, see Perello and Dickson, 2015). The food reward regulation affects both food-seeking behavior and motivation as well as the subsequent hedonic response.

Two experimental papers from the past decade have linked visual food cues, ghrelin, and the neurological control of eating behavior. First, Schüssler et al. (2012) showed in humans that viewing food images stimulates ghrelin secretion. This result suggests that the mere sight of food, even in image form, can cause physiologic food anticipation (contra Tooming, 2021). Second, van der Plasse et al. (2013) demonstrated in rats that visual food cues (a simple light signal Pavlovially associated with food availability) activated medio-hypothalamic brain regions, relevant in behavior regulation, and increased food anticipatory activity (i.e., laboratory animal’s analog of food seeking behavior, see Mistlberger, 1994) similarly to exogenously administered ghrelin.

Viewing food photography induces physiological responses similar to seeing real food, yet how do the two stimuli compare? Researchers have not been in full agreement on this point. For instance, Romero et al. (2018) systematically investigated the difference of real and food images as cues to stimulate expected satiety and willingness-to-pay. The researchers concluded that real food and depictions elicit significantly different responses, based on the findings that participants expected real food to be statistically significantly more satiating (+0.55 on a 5-point satiety scale) and were willing to pay 6% more. However, despite the statistical significance, one may say that the absolute differences were relatively small. More to the latter point, a meta-analysis of a total of 3,300 study participants found equal outcome effect sizes in downstream eating behavior in response to viewing real food and food depictions (Boswell and Kober, 2016). Therefore, it seems reasonable to conclude that food photos elicit similar responses as seeing real food, both in kind and degree.

#### The Impact of Food Photography Content

Not all food photography are created equal. The degree to which food photos induce cravings depends on both *what* and *how* it is represented. For instance, researchers have studied the interaction between portion size and actual consumption with both real food and images. Rolls et al. (2002) established that the portion size (of actual food) influences overall food consumption in a meal. Madzharov and Block (2010) replicated the portion size effect by showing how the quantity of food printed on the packaging positively correlates with actual consumption. This would suggest that images that depict larger food quantities elicit stronger cravings. However, two other studies suggest that the effect of portion size is not so straightforward. In one study, by reducing the size of the serving plate while keeping the depicted food portion constant, participants rated the food more appetizing, were willing to pay more for it, yet served themselves *less* of the actual food (Petit et al., 2018). In another study, images of meals judged “too big” lead to lower activation in brain areas relevant in reward processing (Toepel et al., 2015). It seems, then, that the perception of portion sizes depends both on the depicted food quantity and the presentation, and that greater depicted portions lead to increased food intake only up to a point.

In the previous section, we have shown evidence for the fact that consumption of food can decrease the attentional bias toward visual cues of those same foods. Food photos also seem to induce such sensory-specific satiation. In an experiment by Larson et al. (2014), study participants that had viewed 60 salty images rated subsequently consumed peanuts as less appetizing, compared to participants who had viewed 60 sweet images or only 20 images of either taste. Similar work in the context of mental imagery suggests that this visually induced satiety should translate into actually decreased consumption (see Morewedge et al., 2010).

We have seen that the brain can automatically infer the energy-content of depicted foods. However, given that healthiness evaluations affect both food choice (Nikolova and Inman, 2015) and portion size (Suher et al., 2016), how do consumers determine the healthiness of depicted food? One common way is for consumers to simply categorize food as either “good” or “bad” (Rozin et al., 1996). A more subtle way is by how pretty the food looks. According to earlier research, food naturalness is heuristically linked to healthiness (Rozin, 2005). Following up on these results, Hagen (2020) conducted a series of experiments showing that consumers consider prettier images of the same food as more natural and, hence, more healthy, with an increased willing-to-pay. However, only images depicting food arranged according to classic esthetics, e.g., in symmetry and regular, induced the perception of naturalness. Also more generally, consumers seem to prefer symmetrically arranged of food depictions (Velasco et al., 2016).

Going beyond the physical food itself, food photographs can be manipulated with image filters. To study the effect of image filters on consumer engagement, measured in the form of views, likes, comments, and favorites, Flickr engineers analyzed of 4.6 million images of the platform (Bakhshi et al., 2019). On a general note, the engineers found that food photos had an approximately 30% higher engagement-likelihood, compared to non-food photos. Filter-edited food images were 16% more likely to be viewed, compared to raw images. It is important to remark that this was a correlational study and, while accounting for a multitude of variables, the authors emphasize that their analysis does not prove causality. To empirically study the impact of color on food image attractiveness, Paakki et al. (2019) asked consumers to rate the attractiveness of 10 different salads, five pale and five colorful. The color of each salad was measured with a colorimeter, and consumers ranked the photos of the salads according to attractiveness, and provided their reasoning as free text. The quantitative analysis showed that consumers preferred the colorful salads with high color contrast and saturation, as well as salads with the complementary colors red and green. Furthermore, text analysis suggested that the colorful salads signaled freshness and ingredient diversity. At least for fruit and vegetables, we may, therefore, conclude that higher contrast and deeper color saturation make for more attractive food photos.

In the introduction, we have alluded to the fact importance of sociality, both generally and in relation to food. It, therefore, does not seem overly surprising that depicted social cues also modulate the interaction between viewing food images and eating behavior. Indulging in unhealthy food typically causes a cognitive conflict between short-term indulgence and long-term health maintenance. This conflict reduces available cognitive resources and leads to reduced taste evaluations (Poor et al., 2013). Images of others eating (so-called “consummatory” images) unhealthy food serve as evidence for indulgence’s social acceptability. This evidence resolves the cognitive conflict and increases taste evaluations (Poor et al., 2013). Interestingly, this need not even be images of others eating – self-reflections and statics self-portraits during eating also increase taste evaluation and consumption (Nakata and Kawai, 2017). Hence, images of unhealthy food cueing the consumption’s social acceptability seem to promote unhealthy eating behavior more than depicting the food without any social cues.

#### The Impact of Photo Composition and Interaction Medium

The ease of mental simulations is a crucial factor for triggering downstream effects such as desire to eat. The photograph composition, as well as the interaction medium, make simulation easier if they mimic natural interaction with the food. For example, research by Shen et al. (2016) demonstrated that directly interacting with food images through touchscreens, rather than indirectly through a mouse or a touchpad, biases food choice toward indulgent food and away from healthy food. The touchscreen allows for a more direct and natural interaction with the food image. This triggers a “grabbing” reflex, subsequently increasing the desire for the hedonic, i.e., inherently pleasurable, product.

A photo compositional detail that influences the ease of mental simulation is the orientation of a dish. In one scenario studied by Elder and Krishna (2012), a plate of cake had a fork either on the left or the right side. As might be predicted, right-handed participants rated the image with the fork on the right side higher in terms of wanting and willingness-to-buy, and vice-versa for left-handed participants. Apart from orientation, the perspective of the image also seems to influence eating behavioral outcomes. Christian et al. (2016) found that imagining the consumption of indulgent food from a first-person perspective increased consumption and willingness-to-pay more than imagining it from a third-person perspective. The authors concluded that the ease of mental simulation makes the first-person perspective more affectively stimulating. In contrast, the simulation difficulty in the third-person perspective and the concomitant reduction in affect allows for more cognitive and deliberate decision making. These results were replicated in a neuroimaging study by Basso et al. (2018) in the context of food videos. The fMRI data suggest that a first-person perspective leads to higher activation in brain areas relevant for food pleasure and reward processing. In neither study did perspective influence outcomes of viewing healthy food.

Other aspects of perspective also seem to affect photo attractiveness and, presumably, the downstream behavioral response. In the context of developing real-time applications estimating food photo attractiveness, researchers from Japan have systematically assessed the effect of photography angle, scaling, and blurring of food photography. Albeit food photos were rated by only a small number of human raters,^2^ and the findings certainly do not come as a surprise to professionals (see, e.g., Glyda, 2019), the researchers nevertheless document that the vertical photography angle (Takahashi et al., 2017) as well as *post-hoc* blurring and scaling (Hattori et al., 2018) impact the attractiveness of food photos. Furthermore, marketing of such assistive technology to consumers might soon make creating appetizing food photos trivial.

### Creating Food Photography

Like any other behavior (cf. Gibson, 1979), eating is goal-directed. One of the implicit consumption goals is eating pleasure (cf. Poor et al., 2013). We suspect that the effects of photography on eating behavior depend on the congruence between the salient consumption goal and behavior (for a review on goal-priming, see Papies, 2016). Incongruence can lead to a cognitive conflict, which reduces the intensity of experienced pleasure (cf. Poor et al., 2013). Coary and Poor (2016) have shown that food photography can increase the taste evaluation of indulgent but not healthy food. The researchers provide evidence illustrating how photography both (1) directs attention to the food, i.e., increasing awareness of its properties, and (2) delays consumption, leading to an increased savoring of the indulgent experience. Healthy food is inherently less tasty than unhealthy food (Raghunathan et al., 2006), so healthy eating is incongruent with the consumption goal of pleasure. However, when Coary and Poor made descriptive healthy (vs. unhealthy) eating social norms salient, photographing healthy food before consumption lead to the same increase in taste evaluations seen previously for indulgent food. The awareness of the healthy eating social norm changed the consumption goal from indulgence to healthy eating, thus aligning goal and behavior. Photography becomes an amplifier for goal-dependent reward. One might ask, if being on a diet would shift the consumption goal toward healthy eating and enable photography’s pleasure-enhancing effect *by default*? (cf. Papies and Hamstra, 2010).

In agreement with the findings by Coary and Poor, Diehl et al. (2016) show that photography, in general, increases the engagement in and enjoyment of experiences. The researchers approached customers at a highly frequented food market, asking them to participate in a questionnaire about their meal – either with or without instructing participants to take photographs of their meal. Customers that photographed their meal reported higher engagement and consequently increased meal enjoyment. The researchers also provide evidence for the claim that photography generally amplifies the inherent valence of experiences – positive experiences become more positive and negative experiences more negative. The *intention* to photograph directs attention toward valuable visual aspects of an experience – no need to actually photograph anything. Especially in the context of food, it might be worth noting that directing attention *toward* the visual aspects also means directing it *away* from other sensory aspects (cf. Barasch et al., 2017).

An important detail that Diehl et al. (2016) highlight is that photography only increases engagement and enjoyment of experiences when it does not interfere with the experience itself. In one of their studies, the researchers varied the interference level of photography. In the fully virtualized experimental setup, the low-interference group had to click a computer mouse to take a snapshot of a museum video. The medium-interference group had to drag and align a virtual camera onto the video, and the high-interference group additionally allowed participants to delete unsatisfactory snapshots. As predicted, the experimental groups differed in their level of enjoyment of the museum video tour, with the low-interference group enjoying the experience most. However, all of the experimental conditions were at least as enjoyable as the no-photography control condition. In the context of food photography sharing, this might mean that using the phone to take a photo of the dish adds to the meal experience, whereas navigating to the Instagram app, editing the image, and posting it detracts from the experience. Maybe, then, it would be better to wait until after the meal to share the experience.

Other researchers have recently looked to compare the effect of food photography and non-food photography on subsequent eating behavior (Yong et al., 2020). The researchers employed a within-participants design across two sessions separated by 1 week. In the first session, participants took photos before starting to eat, either of their food or of non-food objects, with the whole session lasting 12 min. The second session was an exact replication of the first, yet without any photography. The results showed no difference between the two photography conditions in any outcome measures, including ad-libitum food consumption, meal enjoyment, liking, wanting, or willingness-to-pay. Ad-libitum consumption in the first session was lower than in the second session. Due to the study design, it is impossible to say whether the fixed session order affected the results. Moreover, the lack of a comparable control group also raises the question, whether the similarity of the photography conditions is due to a general null finding or their actual equivalence. Furthermore, the study at hand took place in a laboratory setting, compared with the previous study’s food market. In the former, baseline attention might have already been optimal, whereas, in the latter, the distracting environment made photography beneficial. The authors justify their study design referencing previous literature (i.e., Coary and Poor, 2016; Diehl et al., 2016), yet the lack of a proper control group or session randomization seem like a missed opportunity.

Although the above-mentioned literature has investigated food photography in terms of relevant end-points, such as food intake and meal enjoyment, research has yet to look at the activity mechanistically. Such studies in physiology, biometrics, and neurology may shed further light on the theoretical and practical interaction of food photography and eating behavior.

### Sharing Food Photography

We have previously seen that social cues and norms can determine how viewing and creating food photography influences food intake and meal enjoyment. These results illustrate the social component of food. Therefore, it seems appropriate to investigate how interacting socially with and through food photography affects eating behavior. However, to our knowledge, the interaction of sharing food images and eating behavior has yet to be studied quantitatively. A potential explanation for the dearth of direct research on food photography sharing could be that researchers may consider observer effects more interesting based on the assumption that more people view food images than create and share them. In this section, we will, therefore, draw mostly on sociological research, as well as media reports, to outline what kind of food photography is shared, why it is shared, and how it might influence the sharer herself. In later parts of this review, we will expand on the topic by relating analogous scientific research to hypothesize about the impact of food photography sharing on eating behavior.

Social support is one reason to share food photography. People are known to use their Instagram profiles to track eating behavior, i.e., as food diaries (Connolly, 2015; Chung et al., 2017). People also use Instagram to supplement the recovery from eating disorders (Mirhashem, 2015; Benveniste, 2016). Using the platform as a food journal and recovery report, people can digitally receive emotional support from peers. However, Instagram supposedly invites and amplifies the natural tendency for social comparison and, as such, the maintenance of a favorable public presence can overtake the original intended purpose of supporting recovery.

The latter claim is further supported by sociological evidence. To uncover the motivations for food photo sharing, Atwal et al. (2019) conducted a diary- and focus group-study of French Fine Dining consumers. The researchers found that photos were intended for both private and public sharing. Motivations could be categorized as either experiential or symbolic. The experiential motivation revolved around enhancing the hedonic meal experience, altruism through sharing of information for others, and passion-collection. Symbolic motivations meant seeking social status, uniqueness, building self-esteem, or to present oneself socially favorably. These findings roughly correspond with earlier findings of Wang et al. (2017), who had investigated the motivations of traveler’s food photography sharing.

In their (n)ethnographic study of online food photography sharing, Kozinets et al. (2016) found that the digital realm also affords the possibility of infinite food sharing and consumption. For some study participants, the practice was an explicit replacement (or compensation) for (the nowadays maybe rare) in-person commensality. The researchers also identified three levels of food-related social network participation: private, public, and professional. The overall distinction between these levels is, to paraphrase the researchers, that the private levels discipline, while the public ones exacerbate, food-related passions.

The content shared at the private level is at least as much social as it is about the food itself. For example, people themselves might be part of the photo (for public- and professional content this is not the case). Here, social norms also have a strong influence on behavior, at least in terms of sharing content. The researchers describe a mostly passion-repressive effect of these social norms. The question is how big this digitally-induced repressive effect is on actual eating behavior – will people change their behavior or simply omit “sinful” indulgences from being documented? As for now, we do not know.

As network participation becomes increasingly public and, ultimately, professional, content promotes “a concentrated state of pure consumption” (contra Tooming, 2021) and competes for audience attention. While content at the public level aims to shape and signal and image of the sharer herself, as a mechanism to build new relationships (cf. also Chen, 2017), professionals seek cultural influence and audience-engagement. The competition for attention drives a sort of “food porn extremism,” resulting in transgressive content that appeals to the universal, primal, and visceral instincts of the audience (cf. also Matthen, 2015). This trend appears to stand in opposition to mainstream global obesity narratives promoting healthy eating and caloric austerity. Evidence for this notion of defiance could be seen in the paucity of *“broccoli or salad porn”* (Kozinets et al., 2016, p. 675). While not explicit in the aforementioned work, the implication in terms of the content creator’s own eating behavior might be that in seeking for attention-catching transgressive novelty, she herself could fall prey to the bait.

There are further reasons to criticize the pervasive nature of food photography on digital platforms. The ubiquity of food photography is seen as a symptom – and by extension, amplifier – of societies and individuals’ generally unhealthy relationship with food.^3^ The obsession with photography supposedly devalues the multisensory experience – including flavors, atmosphere, and social aspects – in favor of a single-minded focus on the visual (Kingkade, 2013; Rawlinson, 2020).^4^ This conceptualization seems to imply a zero-sum game – if vision is at the center of attention, and then all other senses must be at the periphery and, therefore, less important. Furthermore, the habit of sharing food photography proposedly changes what people eat, and changes even the primary motivation behind eating (see generally Turner, 2020; Turner and Gilmore, 2020). According to these authors, some people have come to choose food *solely* based on visual appeal and eat for social recognition instead of for taste and nourishment. In other words, the commentators lament precisely the fact that food “has moved beyond simply fuel” (Kingkade, 2013).

We will return to some of the above-mentioned themes in the next section to discuss future perspectives.

### Interim Summary

So far, we have reviewed the literature on how different aspects of digital food photography, i.e., viewing, creating, and sharing, might influence our real-life eating practices (cf. Figure 1). Viewing food photos elicits similar responses to seeing real food (Boswell and Kober, 2016). Viewing a few food images can stimulate appetite (van der Laan et al., 2011), while viewing many images may induce satiety (Larson et al., 2014). These effects depend on the mental simulation of eating the depicted food. To this end, differences in image composition can make a substantial difference for the extend or ease of mental simulation (e.g., Elder and Krishna, 2012; Basso et al., 2018). Besides, images containing social cues to indulge influence food intake (cf. also Coary and Poor, 2016; Nakata and Kawai, 2017), as they can be used to, presumably unconsciously, justify such behavior.

The intention to photograph itself can increase the eater’s attention on the food and amplify the pleasure of eating (Coary and Poor, 2016; Diehl et al., 2016; contra Yong et al., 2020). However, this effect seems to also depend on the consumption goal (Poor et al., 2013; Coary and Poor, 2016), for example, whether people, at least implicitly, eat for pleasure or for health. Only when the meal is congruent with the consumption goal does photography lead to increased enjoyment (Coary and Poor, 2016).

Finally, researchers have not yet studied the eating behavioral outcomes of food photography sharing, *per se*. Following anecdotal reports, the habit can be used in food diaries to improve eating behavior and aid recovery from eating disorders (Mirhashem, 2015; Benveniste, 2016). Sociological evidence suggests that consumers mainly share food photography for social reasons, while professionals seek attention for their work, thus proliferating the online space with highly appetizing food photos. To this end, food photography sharing has also been criticized for distorting the value placed on non-visual meal aspects (Kingkade, 2013) and changing the primary motivation for eating in the first place (Turner and Gilmore, 2020).

Next, we will draw upon scientific literature to outline several future research avenues related to the current trend of digital food photography (cf. Table 1). First, we examine the potential danger of viewing the endless variety of appealing food images offered on social media and how common usage patterns may lead to a net increase in appetite and, ultimately, food intake. Hereafter, we review the supporting evidence for encouraging photography at the table for increasing both food pleasure and improve long-term food intake regulation. Finally, we draw upon research from social eating to hypothesize how sharing food photography over social media may influence eating behavior. Table 1 summarizes the evidence of demonstrated effects of digital food habits on eating behavior as reviewed in previous sections, and the predicted effects we will discuss in subsequent sections.

**Table 1:** Published research of digital food habits, mediating mechanisms, and their (predicted) effects on eating behavior.

Food photography activity	Outcome (predicted)		Mechanism	References
Viewing	Food intake	↑	Visual stimulation of hunger	van der Laan et al., 2011; Pursey et al., 2014; Boswell and Kober, 2016
			Depicted portion size	Madzharov and Block, 2010; Toepel et al., 2015
			Depicted social cues	cf. also Poor et al., 2013; Nakata and Kawai, 2017
		(↑)	Depicted food variety	cf. Rolls et al., 1981a; Galak et al., 2009
			Mental simulation optimized image composition	cf. Christian et al., 2016; Basso et al., 2018
		↓	Image-induced sensory-specific satiety	Larson et al., 2014; but cf. Missbach et al., 2014
	Wanting	↑	Mental simulation optimized image composition	Elder and Krishna, 2012; cf. Christian et al., 2016; Basso et al., 2018
			Esthetic plating	cf. Velasco et al., 2016; Hagen, 2020
	Healthy food choice	↓	Grabbing reflex triggered on touchscreen devices	Shen et al., 2016
		(↓)	Depicted social cues	cf. Poor et al., 2013
		(↑)	Esthetic plating	Velasco et al., 2016; cf. Hagen, 2020
Creating	Meal enjoyment	↑	Increased attention	Coary and Poor, 2016; Diehl et al., 2016; contra Yong et al., 2020
		(↑)	Increased sense of personal responsibility	cf. Norton et al., 2012; Dohle et al., 2014
	Healthy food choice	(↓)	Increased sense of personal responsibility	cf. Hagen et al., 2017
	Portion size	(↓)	Increased sense of personal responsibility	contra Dohle et al., 2014; cf. Hagen et al., 2017
	Food intake	(↓)	Increased attention	cf. Robinson et al., 2013, 2014; contra Yong et al., 2020
			Increased food intake awareness	cf. Robinson et al., 2013
			Improved meal-time episodic memory	Higgs and Donohoe, 2011; cf. Robinson et al., 2013, 2014; Barasch et al., 2017
		(↑)	Increased sense of personal responsibility	cf. Dohle et al., 2014
Sharing	Food intake	(↓)	Digitally-mediated social comparison	cf. generally Herman, 2015; cf. Barasch et al., 2018
		(↑)	Digitally-mediated social sharing of responsibility	cf. generally Herman, 2017
	Healthy food choice	(↓)	Fulfillment of identity signaling needs	cf. Grewal et al., 2019
			Digitally-mediated social sharing of responsibility	cf. generally Herman, 2017
		(↑)	Digitally-mediated social comparison	cf. generally Herman, 2017; cf. Barasch et al., 2018
	Meal enjoyment	(↓)	Digitally-mediated social comparison	cf. generally Herman, 2017; cf. Barasch et al., 2018

Parentheses indicate effects that we predict based on related or analogous research.

## Future Perspectives

### Can Viewing Social Media Food Photography Lead to Overeating?

Some researchers have used the fact that viewing a high number of food images can induce sensory-specific satiety (as reviewed in previous sections) to suggest that food porn viewing may be a potentially health-promoting habit (e.g., Petit et al., 2016). Other researchers question this optimism (Missbach et al., 2014). First, these researchers replicated previous findings of mental imagery-induced sensory-specific satiety, whereby imagining eating gummy bears, compared to inserting coins into a laundry machine, decreased subsequent actual consumption of gummy bears. The authors went on to show that mental imagery-induced satiation does not occur when self-regulatory resources are depleted (e.g., when people are tired). The result of this study dramatically diminishes the practical utility of viewing food images to induce satiety, as it would be in precisely these situations of mental fatigue, and thereby impaired self-regulation (cf. Baumeister and Vohs, 2003), where external tools would be most beneficial.

Instagram and similar food-related social media sites are like buffets. Their image feeds are full of large varieties of indulgent foods, often depicting large portion sizes. The previous section on viewing food photography has established that type of food and the presented portion sizes are problematic, as they encourage overconsumption. Variety in a meal has a similar effect on food intake. Early research has established that within-meal food variety increases overall energy intake (Rolls et al., 1981a; see, e.g., McCrory et al., 2012 for a more recent review). This effect is closely related to sensory-specific satiety (see Remick et al., 2009). Moreover, the effect of variety also applies to mental imagery. Galak et al. (2009) instructed study participants to eat until satiated and subsequently recall the variety of past eating experiences. The recall “recovered” participants from satiation, thus, they would continue eating. While the effect of food *image* variety on food intake has yet to be studied, it seems natural to assume that the same overall effect would be seen, given the established connection of visual food cues and mental simulation. In combination, the effects of depicted food type, variety, and portion size suggest that food image platforms such as Instagram provide optimal appetite stimulation, which may sabotage any individual’s best effort to control their food intake.

Overall, for individuals concerned about overeating, it seems justified to avoid food porn viewing, especially on social media on the smartphone.^5^

### Should Food Photography Be Encouraged?

#### Food Photography to Enhance Agency

Agency is the sense of being in control of one’s action, and is, therefore, an important motivator of goal-directed behavior (Bandura, 2010). Expenditure of effort is known to increase agency (Demanet et al., 2013). In a series of experiments, Hagen et al. (2017) have shown that low physical involvement when obtaining food decreases the sense of responsibility and concomitantly leads to less healthy food choices. The authors propose that the mechanism behind this result is due to self-serving (re)attribution – the psychological tendency to interpret behavior so that the most beneficial self-conception can be maintained (e.g., Heider, 1958). Thus, it is common for people to assume agency for their own positive behavior and reject agency, i.e., point at external factors, for negative behavior. Facts only loosely constrain such interpretations of personal responsibility. However, as these interpretations need to be socially believable, they hinge on the availability – or absence – of “reasonable” evidence. The studies of Hagen et al. (2017) show that comparatively minor physical actions are sufficient evidence to change the sense of agency and, consequently, behavior. For example, in a waiting room setting, having participants serve themselves unhealthy snacks from a big jar, compared with having pre-filled cups available, was sufficient to reduce sweets consumption dramatically. Follow-up investigations showed that choosing unhealthy food in low physical involvement conditions impacts positive self-regard less negatively than high physical involvement conditions. In contrast, when serving healthy food, there were no differences in amount consumed or feelings of positive self-regard between physical involvement conditions. We hypothesize that photographing food represents reasonable evidence of responsibility for the food choice and portion size, comparable to increasing physical involvement as reported by Hagen et al. (2017). As such, food photography could enhance the negative impact that unhealthy eating has on self-regard and, therefore, increase the likelihood of making healthier eating decisions long-term. The sense of agency also influences people’s value perception. Norton et al. (2012) have shown that, mediated by an increased sense of agency, physical involvement increases the valuation of creations – the so-called “IKEA effect.” Self-cooked food also tastes better, yet also leads to increased consumption (Dohle et al., 2014). These results are congruent with the digital photography-mediated increase in meal enjoyment reported by Diehl et al. (2016). By analogy, the congruence between these findings from the “physical” and the “digital” world suggests that food photography could lead to similarly increased healthy eating choices as seen in Hagen et al. (2017). In sum, we propose food photography as a promising method to increase people’s sense of responsibility and, thereby, make healthier food choices.

#### A Photographic Food Diary to Promote Food Intake Memory

Food intake memory is inaccurate, with a self-serving bias toward underreporting (Lichtman et al., 1992; Schoeller, 1995; Seale and Rumpler, 1997; Hill and Davies, 2001). Food photography could be a more objective way to record eating behavior. The practice has been validated as a very accurate food intake measurement method in multiple settings and populations (for a review, see Martin et al., 2014). The Rapid Food Photography Method (RFPM) is a well-defined clinical methodology for measuring food intake under free-living conditions. Under 6 days of free-living conditions, the RFPM underestimated daily energy intake by an average of 150 calories, compared to the gold-standard method of Doubly Labeled Water (Martin et al., 2012). The method underestimated energy intake by 17 calories in a single buffet-style meal, compared with weighing the food. To provide some perspective, food labels in the European Union only need to be +/−20% accurate.^6^ Participants of studies in free-living conditions scored the method highly in terms of practical viability. While the RFPM is more intricate than presented here, the point is that photography can provide accurate information of both food quality and quantity.

Carter et al. (2013) have shown that weight loss assistance apps on smartphones are superior in terms of adherence compared to implementations on websites on paper. Given the ubiquity of camera-quipped smartphones in the developed world (Pew Research Center, 2019), food photography thus shows the potential to be a practical and non-intrusive food intake management method.

Memory seems to be an important component in long-term food intake regulation. For example, amnesiacs may experience satiation, yet eat almost an entire second meal only a few minutes later (Higgs et al., 2008). In a systematic review on attentive eating, Robinson et al. (2013) showed that (1) reduced attention and visual cues during eating moderately affect immediate food intake, yet (2) reduced attention had a large effect on *later* food intake, and (3) episodic memory formation was linked to later food intake. In a follow-up experimental study, eating attentively led to a 30% reduction in later food intake in obese women (Robinson et al., 2014). However, contrary to the result of an earlier study (Higgs and Donohoe, 2011), the reduction in food intake was not mediated through improved memory. In the 2014 study, the authors noted that both the experimental and control groups had achieved high memory scores, presumably reaching a ceiling. In their review, the authors call to investigate practical methods that facilitate attentive eating, meal memory formation, and before-meal recall of prior consumption (Robinson et al., 2013).

A photographic food diary may be such a method! After all, photography increases engagement with and visual memory of experiences (Barasch et al., 2017). This effect is due to the shift in attention toward visual and away from other sensory experiences. As was the case for the findings by Diehl et al. (2016) reviewed previously, it is the intention of photographing that increases visual memory, and not necessarily the act of producing the photography itself. To further illustrate the point of photography’s general effect on cognition, the memory of not-photographed objects was also improved. Here, we would like to reiterate that food photography needs to introduce as little distraction as possible *during the meal itself*. Not only does distraction impair the experience, but it also impairs memory formation (see Robinson et al., 2013) – quite the opposite of what we are trying to promote.

The photographic food diary would also be a convenient and effective format to facilitate recall. In her study on photography and memory, Henkel (2014) found that retrieval of museum tour memories was more effectively cued with photos than photographed object’s names. Maybe meal companions should also be included among the food photos, as recall is improved when photos include people (Barasch et al., 2016). Following the findings by Robinson et al. (2013), photographs of recent meals should then be reviewed before a meal to recall prior consumption.

In sum, we suggest that the combination of taking food photos and reviewing them before subsequent meals could improve food choice and intake-regulation. These claims should be investigated to elucidate whether a photographic food diary in fact translates into long-term improvements in body weight management and eating behavior.

#### Manipulating Food Memory

It might make some sense to strategically manipulate previously taken food photos to enhance their eating behavioral effect upon review. One approach could be to influence how satiated one *ought* to be. According to the concept of *expected satiety*, satiety does not depend only on the actual nutritional content of food but also on how satiating it is believed or expected to be (Brunstrom et al., 2011). For example, in one study post-ingestive satiety of identical smoothies depended on whether the researchers told participants that the smoothie contained small or large amounts of fruit (Brunstrom et al., 2011). Based on the information given, the participants reflexively *imagined* how satiated they would be after the meal, affecting also their subsequent physiological response. While the effect studied by Brunstrom et al. (2011) was prospective, i.e., the information came before the sensation, under certain circumstances, it might be possible to trigger a similar effect in the reversed order (cf. Loftus and Pickrell, 1995). In the context of a photographic food diary, reviewing computationally enlarged images of the most recent meals might lead to compensation at subsequent eating occasions. This hypothesis is based on the assumption, that people would expect to be full more quickly, believing that previous meals were larger than they actually have been. AR applications that enlarge food sizes in real-time have already been proven to decrease consumption (Narumi et al., 2012), thus partly validating the concept.

Future research should aim to develop applications to make reviewing past meals convenient, while also measuring the effect that this practice has on long-term eating behavior.

### Is Sharing Food Experiences Digitally Comparable to Social Eating?

As mentioned previously, there is a lack of directly focused research on the effect of sharing food photography with others on eating behavior. In the following sections, we draw on multiple research lines to speculate on potential avenues of research and application.

Food photography can increase the enjoyment of eating experiences, as described above. However, the purpose for taking photos can influence the effects on the experience itself. Barasch et al. (2018) showed how merely *intending* to take photos for sharing can reduce the enjoyment of an experience compared to taking photos for personal use. Increased self-presentational concerns mediate this effect. The self-presentational concerns raise anxiety and shift the focus away from the experience and toward the self, subsequently reducing people’s engagement with, and enjoyment of, the eating experience. For the same reason, audience size affects what is shared in the first place (Barasch and Berger, 2014). According to the Barasch and Berger (2014), audiences larger than one trigger self-presentational concerns, resulting in the sharing of less useful and more self-promotional content. In contrast, during one-to-one communication, people focus more on each other and share more useful information. These findings seem similar to those of Kozinets et al. (2016) levels of social network participation in a food context, discussed earlier. Therefore, we suspect that taking images intended for sharing might raise self-presentational concerns and thus reduce the subsequent meal enjoyment.

The abovementioned effects are analogous to the long-established phenomenon of socially facilitated eating, explicitly, eating with strangers. People eat less when eating with strangers – due to self-presentational concerns (Herman, 2015). The opposite is the case when eating in more acquainted settings. Eating with a friendly group of people is associated with significantly larger energy intake at meals. Specifically, the social context enables individuals to share responsibility and thereby rid themselves of individual responsibility. Some researchers have speculated that people arrange social gatherings precisely to engage in guilt-free indulgence (Herman, 2017). Young et al. (2009) found that this social eating effect depends on gender, both of that of the eater’s themselves and their dining companions (see also Salvy et al., 2007). Specifically, women seem to reduce food intake when sharing a meal with men, while dining with other women increases it marginally. For men, the gender of meal companions seems inconsequential for food intake. Thus, it seems to matter who *you* are and with *whom* you share food with.

Sharing food images might have an unexpected catch. Marketing researchers have found that posting about an identity-signaling product decreases actual purchasing behavior (cf. generally Belk, 1988; Grewal et al., 2019). Essentially, posting about such a product fulfills the need to express identity. Food – and diet – are also expressions of identity (Yun and Silk, 2011; see Chuck et al., 2016). Therefore, we hypothesize that sharing, e.g., healthy food might fulfill the need to portray oneself as a healthy person and consequently decrease the need for actually eating healthily. However, the reverse might be equally true: sharing photos of one’s indulgences might trigger the drive to make more healthy eating decisions. Digital confession, so to speak.

Based on the research presented above, it is unclear whether and how sharing food photography privately or on social media would affect eating behavior. The current scientific evidence seems to be ambivalent about the influences on healthy eating. Considering that many individuals are using social platforms to improve their eating habits (Mirhashem, 2015; see Benveniste, 2016), it is an area critical for future research.

## Conclusion

Thus far, the steps within the cycle of food photography (see Figure 1) have been researched to varying degrees (cf. Table 1). Firstly, the subject of *viewing* food photography has accumulated the most direct research. On the one hand, viewing indulgent food images triggers mental simulations and thereby stimulates appetite (van der Laan et al., 2011; Boswell and Kober, 2016). On the other hand, viewing many food images could also induce sensory-specific satiety (Larson et al., 2014). However, we suggest that this approach is unlikely to be of practical benefit for two reasons. One, food variation in meals opposes the effect of sensory-specific satiety (Rolls et al., 1981a), and online image feeds tend to show a wide food variety. Two, the imagery-induced satiety effect depends on cognitive self-control resources (Missbach et al., 2014), which often are the limiting factor limiting self-control in the first place (cf. Baumeister and Vohs, 2003). Interestingly, viewing healthy food photos does not appear to have much of a beneficial effect (Poor et al., 2013; cf. Christian et al., 2016).

Secondly, *creating* photography has been subject to scientific investigation both generally and in the context of food. Research suggests that photography *per se*, irrespectively of the photographed object, increases (visual) attention (Barasch et al., 2017) and the overall enjoyment of experiences (Diehl et al., 2016). These general effects also seem to apply to food and increase meal enjoyment and taste evaluation (Coary and Poor, 2016; Diehl et al., 2016; contra Yong et al., 2020). However, the photography should interfere as little as possible with the eating experience (Diehl et al., 2016; cf. da Mata Gonçalves et al., 2019) – photo editing and uploading might best be delayed until after the meal. Further, we hypothesize that, by going through the effort of taking a photograph of the food, people will feel more responsible for their food choices, leading to greater satisfaction in the moment and healthier eating behavior over time (Norton et al., 2012; Dohle et al., 2014; cf. Hagen et al., 2017). The photography-induced increase in attention might improve memory (Barasch et al., 2017), which could be exploited to improve long-term bodyweight management (cf. Robinson et al., 2013). In this context, we see it as critical to review previous meals’ photos before subsequent meals (Robinson et al., 2013; cf. Henkel, 2014).

Thirdly, the eating behavioral effect of *sharing* food photos has yet to be studied directly. We can only speculate how the sharing of food photography over social media may influence eating behavior. Existing research in commensality and, more broadly, social psychology suggest that food photography sharing could negatively influence food choice and eating pleasure (e.g., cf. Herman, 2017; Barasch et al., 2018). Future research should scrutinize the effects of food photography sharing.

In conclusion, food photography has become an integral part of many people’s lives. Many questions remain about the influence of digital food photography on healthy eating behavior. However, it already seems clear that, if used wisely, photography can help us develop a healthier and more satisfying relationship with food.

## Author Contributions

TA, QJW, and DVB conceptualized the draft. TA wrote the initial draft and prepared the visualizations. QJW and DVB reviewed and edited the draft. All authors contributed to the article and approved the submitted version.

### Conflict of Interest

The authors declare that the research was conducted in the absence of any commercial or financial relationships that could be construed as a potential conflict of interest.

## Footnotes

^1^We will review contrary evidence in later sections.

^2^In the case of Hattori et al. (2018), 20 computer science students in total.

^3^In her talk at the Canadian Obesity Summit 2013, Taylor also shows an arguably more extreme case of visual obsession with food: food tattoos.

^4^“We take pictures of things that are important to us, and for some people, the food itself becomes central and the rest – the venue, the company, et cetera -- is background” (Kingkade, 2013).

^5^Preliminary survey data of young Danish residents suggest that for 80% of respondents, the smartphone is the most frequently used device to view food photography (unpublished).

^6^The *t*-20% accuracy is technically not completely correct, as accuracy is dependent on constituent and concentration range (European Commission, 2011); for the given argument, this oversimplification is sufficient.
